# Editorial Notes

**Published:** 1894-09

**Authors:** 


### Editorial Notes.

WE see from the *Daily Press* that yellow fever has appeared in Baltimore.

DR. CHAS. P. NOBLE has removed to 1637 N. Broad street, Philadelphia.

THE Private Surgical Infirmary of Dr. Chas. S. Biggs will be reopened for patients at Nashville, Tenn., September 15, 1894.

ON November 20, 21, 22 and 23 the Twentieth Annual Meeting of the Mississippi Valley Medical Association will be held at Hot Springs, Ark. A large attendance is expected.

A STATE MEDICAL AND SURGICAL ASSOCIATION has been organized in Jackson, Miss. A membership of one hundred and twenty names was enrolled. The officers are: Temporary President, Dr. J. H. Lucas, of Greenville, and Dr. H. H. Hughes, Secretary.

THE officers of the American Medical Editors' Association for the ensuing year are: President, Dr. John B. Hamilton, of Chicago; Vice-President, Dr. G. M. Gould, of Philadelphia; Secretary, Dr. H. B. Ellis, of San Francisco. The next meeting will be held in Baltimore in May, 1895.

MEETING OF THE SOUTHERN SURGICAL AND GYNECOLOGICAL ASSOCIATION.—The Seventh Annual Session of the Southern Surgical and Gynecological Association will be held in Charleston, November 13, 14 and 15, and promises to be the most successful in the history of the organization. Papers will be presented by the leading surgeons and gynecologists of the South. The medical profession is cordially invited to attend. Dr. Cornelius Kellock, of Cheraw, S. C., is President.

